# Role of extra cellular proteins in gastric cancer progression and metastasis: an update

**DOI:** 10.1186/s41021-020-00157-z

**Published:** 2020-05-15

**Authors:** Mohammad Reza Abbaszadegan, Majid Mojarrad, Meysam Moghbeli

**Affiliations:** 1 Medical Genetics Research Center, Mashhad University of Medical Sciences, Mashhad, Iran; 2 Department of Medical Genetics and Molecular Medicine, School of Medicine, Mashhad University of Medical Sciences, Mashhad, Iran

**Keywords:** Gastric Cancer, Extra cellular matrix, Metastasis, Microenvironment

## Abstract

**Background:**

Gastric cancer (GC) is one of the most common cancers in the world with a high ratio of mortality. Regarding the late diagnosis, there is a high ratio of distant metastasis among GC cases. Despite the recent progresses in therapeutic modalities, there is not still an efficient therapeutic method to increase survival rate of metastatic GC cases.

**Main body:**

Apart from the various intracellular signaling pathways which are involved in tumor cell migration and metastasis, the local microenvironment is also a critical regulator of tumor cell migration. Indeed, the intracellular signaling pathways also exert their final metastatic roles through regulation of extra cellular matrix (ECM). Therefore, it is required to assess the role of extra cellular components in biology of GC.

**Conclusion:**

In the present review, we summarize 48 of the significant ECM components including 17 ECM modifying enzymes, seven extracellular angiogenic factors, 13 cell adhesion and cytoskeletal organizers, seven matricellular proteins and growth factors, and four proteoglycans and extra cellular glycoproteins. This review paves the way of determination of a specific extra cellular diagnostic and prognostic panel marker for the GC patients.

## Background

The local microenvironment has critical role in regulation of cell functions [1]. The extracellular matrix (ECM) as non-cellular component of microenvironment has important roles in tissue dynamic, morphology, and functions [2]. Therefore, ECM aberration can be associated with abnormal behaviors of cells and tissue homeostasis which results in various disorders such as fibrosis and cancer [3, 4]. The proteins, proteoglycans, polysaccharides, and minerals are some of the main components of ECM that shape basement membrane and interstitial matrix [5]. ECM is composed of various proteins such as collagens, laminins, proteoglycans, and remodeling enzymes. Beside the structural roles, the ECM is associated with signaling pathways and growth factors through binding with BMPs, FGFs, hedgehogs, and WNTs [2]. Therefore, aberrant ECM composition can be oncogenic. ECM degrading enzymes including metalloproteinase and serine protease have destructive influences on tissues which highlights the regulatory role of ECM remodeling enzymes during transcriptional, translational, and posttranslational levels [6, 7]. Type I collagen is the most common type in interstitial matrices, whereas type IV collagen is essential in basement membrane [8]. Collagens function as a scaffold to facilitate tumor cell migration [9]. Increased collagens deposition have been reported during tumor formation [10]. Aging decrease and increase collagen deposition and MMP activity, respectively. Abnormal ECM dynamics promote epithelial mesenchymal transition (EMT) via basement membrane disruption using MMPs up regulation which is a critical step during tumor progression [11, 12]. Since the EMT results in cancer stem cell like properties and drug resistance, it facilitates tumor metastasis [13, 14]. Tumor angiogenesis is also another process associated with ECM in which many ECM components such as endostatin, arresten, and hexastatin have inducing or inhibitory angiogenic functions [15]. The G1/S cell cycle progression is also associated with ECM-cell adhesion in which the ECM activate growth factor through ERK signaling [16, 17]. Moreover, the immune cell processes such as infiltration and activation can be affected by ECM [18].

Carcinogens exert their impact through DNA damage, aberrant cellular processes, and microenvironment alteration [19]. The effects of majority of carcinogens are occurred through modulation of tumor microenvironment. Tumorigenesis is associated with activation of various cell types such as stem cells, fibroblasts, and hematopoietic cells which recruit immune cells to produce cytokines [20, 21]. Carcinogens can change the functions of these cells and ECM components [22]. Tissue remodeling is known by changes in expression and degradation of ECMs [23]. Matrix metalloproteinases (MMPs) as the main ECM remodelers can be affected by carcinogens. Chronic exposure to heavy metals, nicotine, and radiation are associated with tissue remodeling [24, 25]. Moreover, some chemicals regulate tissue remodeling through up regulation of growth factors, cytokines, and extracellular proteins in host cells. These alterations in tissue remodeling genes results in tissue architecture changes which promotes tumor progression [26].

Gastric Cancer (GC) is the fourth common and the second leading cause of cancer-related deaths in the world [27]. Distant metastases are observed among a noticeable ratio of GC patients at the time of diagnosis which have not an effective treatment (5-year survival rate is up to 10%) [28–30]. Therefore, introduction of circulating biomarkers can be helpful to improve early detection and prognosis in such patients. Increased protein-specific fragments belonged to the ECM turnover are released into blood following an aberrant ECM remodeling which can be used as circulating biomarkers for diagnostic and prognostic purposes in cancer patients [31]. Since the main reason of distant metastasis is local microenvironment and extracellular matrix, in the present review, we summarize 47 of the significant ECM proteins which have been reported until now among GC patients in the world including 17 ECM modifying enzymes, seven extracellular angiogenic factors, 12 cell adhesion and cytoskeletal organizers, seven matricellular proteins and growth factors, and four proteoglycans and extra cellular glycoproteins (Table 1). Official symbol, official full name, gene ID, and URL of the reported genes in the present study referred from National Center for Biotechnology Information (https://www.ncbi.nlm.nih.gov) are also presented in Table 2.

**Table 1:** All of the ECM related factors involved in gastric cancer progression and metastasis

Gene	Results	Country	population	Year	Reference
**ECM modifying enzymes**					
MMP9	Polymorphism was correlated with invasive phenotype	Japan	177 patients 224 controls	2005	Matsumura [32]
MMP9	MMP9 expression was correlated with stage, depth of tumor invasion, and lymph node involvement	Poland	54 patients	2009	Mroczko [33]
MMP9	increased levels of expression in advanced stage tumors	Serbia	40 patients 11 controls	2009	Dragutinovic [34]
MMP1	Polymorphism was correlated with poorly differentiation	Japan	215 patients 166 controls	2004	Matsumura [35]
MMP1	Polymorphism was correlated with GC risk	China	422 patients 428 controls	2014	Dedong [36]
MMP2	MMP2 expression was correlated with poor survival, stage, and diffuse type	Finland	315 patients	2006	Mrena [37]
MMP14	High levels of serum MMP14 was correlated with poor survival	Finland	240 patients	2018	Kasurinen [38]
MMP7 and LAMC2	MMP7 and LAMC2 expressions were correlated with tumor aggressiveness	Japan	790 patients	2014	Sentani [39]
ADAMTS8, ADAMTS18, and ADAMTS1	Expressions were correlated with stage	Turkey	25 patients	2017	Kilic [40]
TIMP1	TIMP1 expression was correlated with advanced stages, increased depth of invasion, and metastatic lymph nodes	Poland	54 patients	2009	Mroczko [41]
MMP2, MMP24, and MMP25	MMP2, MMP24, and MMP25 increased expression	Mexico	17 patients 22 controls	2014	De la pena [42]
LOX	Over expression was correlated with local tumor relapse	China	184 patients	2018	Peng [43]
LOXL4	Over expression was correlated with tumor size, depth of invasion, stage, and survival	China	379 patients	2015	Li [44]
PLOD2	Expression was correlated with peritoneal metastasis	Japan	179 patients	2018	Kiyozumi [45]
TFPI2	Hyper methylation	China	114 patients	2017	Hu [46]
ETS1	over expression induced mucosal into the sub mucosal tumor invasion	Japan	110 patients	1996	Nakayama [47]
**Extra cellular angiogenic factors**					
BSG	Expression was correlated with tumor size and lymph node metastasis	Japan	234 tumors 85 normal	2006	Zheng [48]
MMRN2, EMILIN1, and EMILIN2	High expression of MMRN2 in normal gastric mucosa along blood vessels, while the EMILIN2 and EMILIN1 expressions were limited to the lamina propria.	Italy	51 patients	2018	Andreuzzi [49]
HPSE	Polymorphism was correlated with survival	China	155 patients 204 controls	2010	Yue [50]
CTHRC1	Expression was correlated with depth of invasion	China	116 patients	2012	Wang [51]
CTHRC1	Expression was associated with tumor stage, differentiation, survival, and prognosis, depth of invasion, lymph node involvement, and tumor size.	China	166 tumors 30 normal	2014	Gu [52]
ECM1	Expression was correlated with tumor relapse, poor survival, and advanced stages.	China	241 patients	2018	Gan [53]
ECM1	Expression was correlated with tumor depth of invasion and stage.	China	77 patients	2014	Wu [54]
**Cell adhesion and cytoskeletal organizers**					
PCDH8	Was correlated with poor prognosis.	China	144 patients	2018	Lin [55]
CLDN4	Expression was correlated with depth of invasion and lymph node involvement	Taiwan	189 patients	2010	Hwang [56]
LLGL1	Was correlated with distant metastasis.	Germany	56 patients	2019	Desuki [57]
FN1	increased serum levels	Turkey	63 patients 30 controls	2016	Tas [58]
FNDC1	Expression was correlated with sex, survival, and stage.	China	90 patients	2018	Ren [59]
ANOS1	Expression was correlated with stage and prognosis.	Japan	237 patients	2016	Kanda [60]
ITGA4	Methylation	Korea	46 patients	2004	Park [61]
ICAM1 (CD54)	Expressions were correlated with stage and lymph node involvement.	China	84 patients	2014	Dong [62]
CFL1	Over expression	China	33 patients	2017	Wang [63]
HMMR (CD168)	Inverse correlation with lymph node involvement.	Japan	196 patients	2011	Ishigami [64]
MFAP2	Expression was correlated with depth of invasion, lymph node involvement, tumor stage, and poor overall survival	China	168 patients	2018	Wang [65]
SPON2	Expression was correlated with grade of tumor differentiation, depth of invasion, lymph node involvement, and advanced stages.	China	174 patients	2017	Jin [66]
**Matricellular proteins and growth factors**					
SPARC	Was correlated with serosal invasion and poor survival.	Japan	227 patients	2013	Sato [67]
SERPINE1 AND SPARC	Expressions were correlated with poor survival.	China	293 tumors 196 normal	2018	Liao [68]
SPARCL1	Expression was correlated with prognosis, survival, size, and grade.	China	1072 patients	2012	Li [69]
FGFR2 AND THBS1	Over expressions	China	53 patients	2017	Huang [70]
LTBP2	Expression was correlated with depth of invasion, stage, lymph node involvement, poor survival.	China	174 patients	2018	Wang [71]
POSTN	Inverse correlation between expression and stage.	China	25 tumors 8 normal	2014	LV [72]
**Proteoglycans and extra cellular glycoproteins**					
VCAN	Expression was correlated with poor prognosis.	China	101 patients	2015	Shen [73]
ASPN	Over expression.	China	46 patients	2015	Ding [74]
LUM	Up regulation in gastric CAFs was correlated with advanced stage, poor survival, depth of invasion, and lymph node involvement.	China	117 patients	2017	Wang [75]
MUC2	Was correlated with grade.	Egypt	28 patients	2011	Khattab [76]

**Table 2:** ECM related genes studied in GC patients

Official symbol	Official full name	Gene ID	URL
ADAMTS1	ADAM metallopeptidase with thrombospondin type 1 motif 1	9510	https://www.ncbi.nlm.nih.gov/gene/9510
ADAMTS18	ADAM metallopeptidase with thrombospondin type 1 motif 18	170,692	https://www.ncbi.nlm.nih.gov/gene/170692
ADAMTS8	ADAM metallopeptidase with thrombospondin type 1 motif 8	11,095	https://www.ncbi.nlm.nih.gov/gene/11095
ANOS1	Anosmin 1	3730	https://www.ncbi.nlm.nih.gov/gene/3730
ASPN	Asporin	54,829	https://www.ncbi.nlm.nih.gov/gene/54829
BSG	Basigin (Ok blood group) (EMMPRIN)	682	https://www.ncbi.nlm.nih.gov/gene/682
CFL1	Cofilin 1	1072	https://www.ncbi.nlm.nih.gov/gene/1072
CLDN4	Claudin 4	1364	https://www.ncbi.nlm.nih.gov/gene/1364
CTHRC1	Collagen triple helix repeat containing 1	115,908	https://www.ncbi.nlm.nih.gov/gene/115908
CXCR1	C-X-C motif chemokine receptor 1	3577	https://www.ncbi.nlm.nih.gov/gene/3577
CXCR2	C-X-C motif chemokine receptor 2	3579	https://www.ncbi.nlm.nih.gov/gene/3579
ECM1	Extracellular matrix protein 1	1893	https://www.ncbi.nlm.nih.gov/gene/1893
EGFR	Epidermal growth factor receptor	1956	https://www.ncbi.nlm.nih.gov/gene/1956
EMILIN1	Elastin microfibril interfacer 1	11,117	https://www.ncbi.nlm.nih.gov/gene/11117
EMILIN2	Elastin microfibril interfacer 2	84,034	https://www.ncbi.nlm.nih.gov/gene/84034
ETS1	ETS proto-oncogene 1, transcription factor	2113	https://www.ncbi.nlm.nih.gov/gene/2113
FGF7	Fibroblast growth factor 7	2252	https://www.ncbi.nlm.nih.gov/gene/2252
FGFR2	Fibroblast growth factor receptor 2	2263	https://www.ncbi.nlm.nih.gov/gene/2263
FN1	Fibronectin 1	2335	https://www.ncbi.nlm.nih.gov/gene/2335
FNDC1	Fibronectin type III domain containing 1	84,624	https://www.ncbi.nlm.nih.gov/gene/84624
FOXC2	Forkhead box C2	2303	https://www.ncbi.nlm.nih.gov/gene/2303
HMMR	Hyaluronan mediated motility receptor (CD168)	3161	https://www.ncbi.nlm.nih.gov/gene/3161
HPSE	Heparanase	10,855	https://www.ncbi.nlm.nih.gov/gene/10855
ICAM1	Intercellular adhesion molecule 1 (CD54)	3383	https://www.ncbi.nlm.nih.gov/gene/3383
ITGA4	Integrin subunit alpha 4	3676	https://www.ncbi.nlm.nih.gov/gene/3676
LAMC2	Laminin subunit gamma 2	3918	https://www.ncbi.nlm.nih.gov/gene/3918
LGALS1	Galectin 1	3956	https://www.ncbi.nlm.nih.gov/gene/3956
LLGL1	LLGL scribble cell polarity complex component 1	3996	https://www.ncbi.nlm.nih.gov/gene/3996
LOX	Lysyl oxidase	4015	https://www.ncbi.nlm.nih.gov/gene/4015
LOXL4	Lysyl oxidase like 4	84,171	https://www.ncbi.nlm.nih.gov/gene/84171
LTBP2	Latent transforming growth factor beta binding protein 2	4053	https://www.ncbi.nlm.nih.gov/gene/4053
LUM	Lumican	4060	https://www.ncbi.nlm.nih.gov/gene/4060
MFAP2	Microfibril associated protein 2	4237	https://www.ncbi.nlm.nih.gov/gene/4237
MMP1	Matrix metallopeptidase 1	4312	https://www.ncbi.nlm.nih.gov/gene/4312
MMP14	Matrix metallopeptidase 14	4323	https://www.ncbi.nlm.nih.gov/gene/4323
MMP2	Matrix metallopeptidase 2	4313	https://www.ncbi.nlm.nih.gov/gene/4313
MMP24	Matrix metallopeptidase 24	10,893	https://www.ncbi.nlm.nih.gov/gene/10893
MMP25	Matrix metallopeptidase 25	64,386	https://www.ncbi.nlm.nih.gov/gene/64386
MMP7	Matirix metallopeptidase 7	4316	https://www.ncbi.nlm.nih.gov/gene/4316
MMP9	Matrix metallopeptidase 9	4318	https://www.ncbi.nlm.nih.gov/gene/4318
MMRN2	Multimerin 2	79,812	https://www.ncbi.nlm.nih.gov/gene/79812
MUC2	Mucin 2, oligomeric mucus/gel-forming	4583	https://www.ncbi.nlm.nih.gov/gene/4583
MUC6	Mucin 6, oligomeric mucus/gel-forming	4588	https://www.ncbi.nlm.nih.gov/gene/4588
NODAL	Nodal growth differentiation factor	4838	https://www.ncbi.nlm.nih.gov/gene/4838
PCDH8	Protocadherin 8	5100	https://www.ncbi.nlm.nih.gov/gene/5100
PLOD2	Procollagen-lysine,2-oxoglutarate 5-dioxygenase 2	5352	https://www.ncbi.nlm.nih.gov/gene/5352
POSTN	Periostin	10,631	https://www.ncbi.nlm.nih.gov/gene/10631
SERPINE1	Serpin family E member 1	5054	https://www.ncbi.nlm.nih.gov/gene/5054
SPARC	Secreted protein acidic and cysteine rich	6678	https://www.ncbi.nlm.nih.gov/gene/6678
SPARCL1	SPARC like 1	8404	https://www.ncbi.nlm.nih.gov/gene/8404
SPON2	Spondin 2	10,417	https://www.ncbi.nlm.nih.gov/gene/10417
TFPI2	Tissue factor pathway inhibitor 2	7980	https://www.ncbi.nlm.nih.gov/gene/7980
THBS1	Thrombospondin 1	7057	https://www.ncbi.nlm.nih.gov/gene/7057
TIMP1	TIMP metallopeptidase inhibitor 1	7076	https://www.ncbi.nlm.nih.gov/gene/7076
TIMP3	TIMP metallopeptidase inhibitor 3	7078	https://www.ncbi.nlm.nih.gov/gene/7078
VCAN	Versican	1462	https://www.ncbi.nlm.nih.gov/gene/1462

## Main text

### ECM modifying enzymes

In this section, we summarize 17 gene products including seven matrix metallopeptidases (MMP1, MMP2, MMP7, MMP9, MMP14, MMP24 and MMP25), laminin subunit gamma 2 (LAMC2), three ADAMTS metallopeptidases (ADAMTS1, ADAMTS8 and ADAMTS18), 2 TIMP metallopeptidases (TIMP1, TIMP3), three lysyl oxidase (LOX, LOXL4, and PLOD2), and tissue factor pathway inhibitor 2 (TFPI2).

Matrix metalloproteinases (MMPs), heparanase, cathepsins, urokinase plasminogen activator, and lysyl oxidase are ECM modifying enzymes contributing with tumor metastasis [77–79]. Degradation of ECM is mainly mediated by MMPs which are zinc dependent endopeptidases secreted by various cells such as fibroblasts and endothelial cells. Their activation is depended to the calcium and neutral pH [80, 81]. MMPs are categorized based on their substrate and cellular location into four groups including collagenases, stromelysins, gelatinases, and membrane-associated MMPs. Regulation of ECM through MMPs has a key role in tumor progression and metastasis [82]. Beside other proteases, the MMPs can also activate variety of factors such as tyrosine-kinase receptors, growth factors, and cell-adhesion molecules [7, 80]. Type IV collagen is one of the main components of basal membranes and ECM which can be degraded via MMP2 and MMP9 [83]. The 1562C/T promoter polymorphism of MMP9 has been assessed in a sub population of Japanese GC cases which showed higher frequency of T allele in advanced-stage GC cases. Moreover, T allele significantly increased the lymph node involvement [32]. Another study has been reported that there were correlations between MMP9 expression, stage, depth of tumor invasion, and lymph node involvement which suggested the MMP9 as a marker of GC metastasis [33]. MMP9 had significant increased levels of expression in advanced stage tumors in comparison with early gastric tumors [34]. MMP1 as a collagenase has a critical role in ECM remodeling. It can be regulated by various transcription factors such as AP1, AP2, and retinoic acid [84]. The 1G/2G promoter polymorphism of MMP1 was assessed in GC patients and showed that there was a significant correlation between 2G allele and poorly differentiation [35]. It has been shown that there was a correlation between rs1799750 and GC in which the patients had significantly higher frequency of 2G allele compared with controls. Moreover, the 2G/2G carriers significantly had higher risk of tumor invasion in comparison with 1G/1G + 1G/2G genotype [36]. MMP2 has a key role in primary steps of tumor progression [85]. Cyclo-oxygenase 2 (COX2) is also associated with inflammation and tumorigenesis that up regulates the MMPs through prostanoids [86]. It has been reported that the epithelial MMP2 expression was correlated with COX2 over expression in GC patients. Moreover, there were correlations between stromal MMP2 expression, poor survival, stage, and diffuse type, whereas the MMP9 was associated with intestinal type [37]. MMP14 is belonged to the membrane-bound MMPs. It has been observed that the serum MMP14 was an independent prognostic factor of GC in which the men with high levels of serum MMP14, pT3–4 tumors, and metastatic lymph nodes had poor survival [38]. MMP7 targets various ECM components such as collagens, elastin, and fibronectin. Laminins are associated with cellular adhesion and differentiation [87].

Laminin subunit gamma 2 (LAMC2) is expressed in tumor cells and can be used as a marker of malignant epithelial cells [88]. The LAMC2 has an epidermal growth factor (EGF)-like domain [89] which can be processed by MMP [90] to interrupt the hemidesmosomes via EGFR of b4 integrin during tumor cell migration. It has been shown that the MMP7, LAMC2, and EGFR expressions were correlated with tumor aggressiveness, advanced T grade, and tumor stage in GC tumors [39].

The human ADAMTS (a disintegrin and metalloproteinase with thrombospondin-like motifs) family of 19 secreted, multidomain proteolytic enzymes is involved in a wide range of biological processes including ECM assembly and degradation, hemostasis, organogenesis and the regulation of angiogenesis [91]. Immunohistochemical assessments have been shown the high expression levels of ADAMTS1, 8, and 18 in GC patients. There were correlations between increased ADAMTS1 and ADAMTS18 expressions and lymph node involvement which introduced them as the markers of lymphatic metastasis in GC patients. The ADAMTS8, ADAMTS18, and ADAMTS1 expressions were also associated with tumor stage [40].

Tissue inhibitors of metalloproteinases (TIMPs) as the main MMPs endogenous inhibitors bind to the enzyme catalytic site [92]. Regulation of basement membranes requires a balance between the functions of MMPs and TIMPs [93]. Up regulation of TIMP1 was reported in various tumors [94]. It has been observed that there was high levels of TIMP1 expression in GC tumors with advanced stages, increased depth of invasion, and metastatic lymph nodes. Moreover, the inflammatory infiltrate cells had higher levels of TIMP1 compared with tumor cells. Therefore, higher levels of expressions in advanced stages can be related to the synthetic role of inflammatory cells as well as tumor cells [41]. Another group has been observed that there were significant higher levels of MMP2, MMP24, and MMP25 expressions in GC compared with SG. Moreover, there were correlations between MMP14, TIMP3, and risk of GC progression [42].

Tumor progression requires networks of both the immediate (cell–cell or cell–matrix interactions) and extended microenvironment (vascularization). The stromal activation is parallel with tumor cell growth and metastasis [95, 96]. Lysyl oxidase (LOX) is an extracellular amine oxidase and regulator of tumor microenvironment through ECM posttranslational modification which promotes tumor cell proliferation [97]. LOX also recruits bone marrow-derived cells which are involved in angiogenesis and immune cells infiltration [98]. It has been reported that the gastric tumor samples had significant higher levels of LOX expression compared with peritumoral samples. Moreover, there was a correlation between LOX over expression and local tumor relapse [43]. LOXL4 over expression was also observed in GC patients which was correlated with tumor size, depth of invasion, lymph node involvement, stage, and poor survival. Moreover, LOXL4 induces gastric tumor cell proliferation and migration through FAK/SRC pathway and ERK phosphorylation. LOXL4 can also increase GC cells adhesion with collagen I and fibronectin which is associated with gastric tumor cell migration and invasion [44]. PLOD2 is a lysyl hydroxylase that hydroxylates lysine in procollagens and is regulated by HIF1 to mediate ECM remodeling [99]. It has been shown that the PLOD2 induces GC progression in hypoxic status via HIF1A and there was a significant association between PLOD2 expression and peritoneal metastasis. The PLOD2 over expressed GC cases had also increased collagen expression in their tumor tissues which showed HIF1A/PLOD2 signaling pathway as a regulator of GC aggressiveness in a collagen-related status [45].

Tissue factor pathway inhibitor 2 (TFPI2) is a serine proteinase inhibitor involved in protection of ECM from degradation [100]. Therefore, loss of TFPI2 increases the aggressive behavior of neoplastic cells [101]. The TFPI2 methylation levels were assessed in gastric tumors compared with corresponding normal margins, which showed TFPI2 hyper methylation in majority of GC tissues. Therefore, they suggested the TFPI2 hyper methylation as an efficient diagnostic marker of GC [46]. Regarding the levels of muscularis mucosa involvement, GC is classified into intramucosal and submucosal types, in which the submucosal tumors have higher rates of lymph node involvement. ETS1 is a transcription factor associated with mesodermal development during embryogenesis [102]. It is involved in tumor invasion through regulation of several genes such as urokinase type plasminogen activator, stromelysin-1, and collagenase-1 [103, 104]. It has been reported that the ETS1 over expression induced mucosal into the submucosal tumor invasion in GC patients. Whereas, there was not any ETS1 expression in normal gastric epithelium. The submucosal carcinomas had higher levels of ETS1 compared with mucosal adenocarcinomas which suggested the role of ETS1 in local invasion to submucosa during GC progression [47].

### Extracellular angiogenic factors

In this section, we summarize seven gene products including extracellular inducer (BSG: EMMPRIN), extracellular angiostatic protein (MMRN2), extracellular matrix glycoprotein (EMILIN2), heparinase (HPSE), collagen triple helix repeat containing 1 (CTHRC1), galectin 1 (LGLS1), and extracellular matrix protein 1 (ECM1).

Angiogenesis is a critical process during tumor growth which initiates from the outgrowth and migration of endothelial cells from parental vessels through vascular endothelial growth factor (VEGF). Cytokines, growth factors, and ECM proteins are important regulators of angiogenesis [105, 106]. In this process the MMPs has a key function in regulation of ECM in endothelial cells to form tube-like structures and blood vessels [107]. The extracellular MMP inducer (EMMPRIN) is a transmembrane immunoglobulin expressed on activated T cells, macrophages, endometrium, keratinocytes, and several solid tumors which induces MMP synthesis [108, 109]. It has been shown that there was a direct correlation between BSG expression, tumor size, and VEGF expression in GC patients. Moreover, the BSG over expressed tumors had higher ability for lymph node metastasis. They concluded that the BSG increases the tumor invasion and angiogenesis through MMP and VEGF up regulations in GC cells [48].

Multimerin 2 (MMRN2) is an extracellular angiostatic protein which suppresses the VEGFA/VEGFR2 signaling pathway [110]. It has been observed that there was high expression of MMRN2 in normal gastric mucosa along blood vessels, while the EMILIN 2 and EMILIN 1 expressions were limited to the lamina propria. The loss of EDEN family significantly affects the treatments efficiency and tumor progression [49]. Heparanase (HPSE) is an angiogenic factor and cleaves heparan sulfate (HS) side chains that are one of the main components of ECM and basement membrane. HS cleavage releases HS-linked angiogenic and growth factors such as FGF and VEGF [111]. Moreover, Heparan sulfate proteoglycans are suggested to induce stem cell properties through WNT and IL6/STAT5 pathways [112]. Therefore, HS and heparanase can be associated with various processes such as cell proliferation, adhesion, migration, and angiogenesis [113, 114]. It has been shown that the GA/AA genotypes of rs11099592 polymorphism were associated with superficial invasion of GC. Moreover, there was a significant correlation between rs6856901 polymorphism of HPSE and survival [50].

Collagen triple helix repeat containing 1 (CTHRC1) is a glycoprotein containing a short collagen triple helix repeat associated with cell migration through regulation of collagen matrix deposition [115]. It also functions in cell response to the arterial injury and vascular remodeling. It has been reported that there was a direct correlation between the levels of CTHRC1 expression and deeper depths of invasion which suggested that the CTHRC1 can be involved in GC progression and metastasis. Promoter hyper methylation has an important role in CTHRC1 silencing. In vitro study also showed that the levels of CTHRC1 expression was associated with TGFB1 promoter demethylation [51]. Another study has been reported that the CTHRC1 up regulation was significantly associated with tumor stage, differentiation, survival, prognosis, depth of invasion, lymph node involvement, and tumor size among a sub population of GC patients [52]. During the metastasis process, tumor cells regulate growth factors, cytokines, ECM, and cancer-associated fibroblasts (CAFs) [116]. Galectin 1 (LGALS1) is a carbohydrate-binding lectin which is up regulated in CAFs and induces angiogenesis [117, 118]. Hh signaling pathway has a critical function during the EMT, angiogenesis, and tumor progression of GC through glioma-associated oncogene 1 (GLI1) [117–120]. The β1 integrin is a cell surface adhesion receptors which is inversely associated with cell adhesion loss and has a lactosamine sequence [121]. It has been shown that the CAFs induce epithelial to fibroblastoid phenotype, decrease E-cadherin, and increase vimentin in microenvironment through secretion of LGALS1 that functions via binding to the lactosamine sequence of β1 integrin [121–123]. Moreover, LGALS1 up regulated the GLI1 in GC cells introducing a key role of LGALS1 in GC metastasis through binding with β1 integrin [122].

Extracellular matrix protein 1 (ECM1) is involved in cell proliferation, migration, angiogenesis, and EMT [124]. During EMT process, cells change their epithelial properties to mesenchymal invasive phenotype via down regulation of E-cadherin and upregulation of vimentin [125]. ECM1 is also associated with glucose metabolism via LDHA, GLUT1, and HIF1A [126]. The ECM1 enhances the EMT and glucose metabolism through up regulation of ITGB4 and activation of FAK/SOX2 signaling pathway. Subsequent, SOX2 up regulation regulates HIF1A, vimentin, β-catenin, and E-cadherin expressions. It has been observed that there were correlations between high ECM1 levels, tumor relapse, poor survival, and advanced stages of tumor among GC patients [53]. Lymphangiogenesis is a critical step during tumor progression that prepares more opportunities for tumor cells to spread using lymphatic system [127, 128]. ECM1 has also an important role in lymphangiogenesis [129]. It has been shown that the GC tumor tissues had significantly higher levels of ECM1 expressions compared with normal margins. There were also significant correlations between ECM1 expression, tumor depth of invasion, and stage [54]. CXCR1 and CXCR2 are IL-8 receptors associated with poor prognosis of GC [130]. CXCR1 is a G protein–coupled receptor involved in signal transduction [131], while CXCR2 is a G protein–coupled receptor associated with inflammation and angiogenesis [132]. It has been shown that there were significant correlations between CXCR1, CXCR2, and MMP9 expressions in which CXCR1 and CXCR2 induce MMP9 expression through JNK/c-JUN and ERK/ETS1 signaling pathways [133–135].

### Cell adhesion and cytoskeletal organizers

In this section, we summarize 13 gene products including a member of the protocadherin alpha gene cluster (PCDH8), laminin subunit gamma 2 (LAMC2), a claudin family member (CLDN4), cellular adhesion proteins (LLGL1 and ANOS1), fibronectin family members (FN1 and FNDC1), integrin alpha chain member (ITGA4), immunoglobulin family [ICAM1 (CD54)], cytoskeletal reorganizer (CFL1), hyaluronan mediated motility receptor (HMMR), microfibril associated protein (MFAP2), and an integrin ligand (SPON2).

Epithelial cell junctions have various functions such as communication, anchoring, and sealing. E-cadherin is the main protein of adherent junctions to stabilize the basolateral cell-cell contact. Therefore, E-cadherin loss increases cell migration [136]. Protocadherin (PCDH) is a member of the protocadherin alpha gene cluster functioning as tumor suppressors or oncogenes. They have an apoptotic role by inhibition of WNT pathway, or induce cell migration and drug resistance [137, 138]. It has been shown that the PCDH8 up regulation was significantly associated with poor prognosis and GC migration through up regulation of LAMC2 [55]. Claudins are the main components of tight junctions in epithelial and endothelial cell membranes which functions as primary barriers and regulate the polarity of epithelial cells [139, 140]. The claudin interacts with variety of proteins such as ZO proteins, RAB3B, PTEN, and ZONAB [141, 142]. It has been shown that there were associations between Claudin 4 (CLDN4) expression, depth of invasion, lymph node involvement, and MMP2,9 expressions which suggested the CLDN4 as a regulator of MMP2 and − 9 expressions [56].

LLGL1 is also a cellular adhesion protein that is involved in epithelial cell polarity and migration through a polarity complex comprising of PAR6, PAR3, PKC, and LLGL [143, 144]. It has been shown that there were significant correlations between LLGL1 loss, distant metastasis, and diffuse type of GC [57]. Fibronectin (FN) as a structural basement membrane glycoprotein has a critical function in cell adhesion, migration, and embryogenesis through cell surface receptors and integrins [145]. It is required for the proper collagen incorporation with ECM [146]. Fibronectin and collagen bind to integrins which are involved in cell–matrix adhesion and signal transduction [147, 148]. Fibronectin is also involved in cell proliferation and migration through mTOR signaling pathway [149, 150]. As a mesenchymal marker, it promotes TGFβ during EMT [151]. FN1 is also activator of STAT3 and MAPK signaling pathways to promote tumor progression and metastasis [152]. It has been observed that there was increased serum levels of fibronectin among GC patients which can be introduced as a diagnostic marker [58]. Fibronectin type III domain containing 1 (FNDC1) contains the type III domain of fibronectin [145]. It has been observed that there was significant higher levels of FNDC1 expression in GC compared with normal tissues. Moreover, there were significant correlations between FNDC1 expression, sex, survival, and tumor stage in GC patients [59].

The Anosmin 1 (ANOS1) is a cell adhesion protein associated with migration of gonadotropin-releasing hormone neurons. It increases the malignant behavior through integrin signaling pathways in brain tumors and promotes cell migration and drug resistance in colon cancer [153, 154]. It has been reported that there were increased levels of ANOS1 expressions in GC cell lines in comparison with normal epithelial cell line which suggested ANOS1 as an oncogene in GC. The GC tissues also showed ANOS1 over expression which was correlated with tumor stage, prognosis, and diffuse type. PCR array also showed ITGAV, FOXC2, and NODAL expressions parallel with ANOS1, while TFPI2 down regulation [60]. ITGAV is a member of the ECM and involved in EMT [155]. The FOXC2 is an important transcription factor during EMT [156]. NODAL is belonged to the TGF-β family which has key role in cell growth and migration [157]. Integrins are cell-surface receptors involving in cell–cell and cell–ECM interactions and tumor metastasis [158, 159]. It has been reported that the ITGA4 expression is lost in the majority of GC cell lines and tumor tissues through DNA methylation [61].

Leptin is an adipokine that is involved in body weight homeostasis through regulation of energy metabolism [160]. Leptin also has a key role in cell migration via up regulation of CD54 in allergic inflammation [161]. ICAM1 (CD54)is belonged to the immunoglobulin family which binds with ITGB2 and ITGAM to facilitate the metastasis and hiding toward immunocytes [162]. It functions as an intercellular adhesion protein and has inhibitory role on tight junction formation in blood-testis barrier during spermatogenesis [163]. Various factors such as RHO and ROCK regulate the ICAM1 expression [164]. It has been shown that the leptin induces GC migration through up regulation of ICAM1 and activation of RHO/ROCK signaling pathway. Moreover, there were correlations between ICAM1 levels, stage, and lymph node involvement in GC patients [62].

Cofilin 1 (CFL1) is a cytoskeletal reorganizer and EMT regulator in tumor cells [165]. It has been shown that there were consistent CFL1 up regulation in GC tissues. TGFB1 induced CFL1 expression in the gastric tumor cells. The lack of CFL1 inhibited actin depolymerization and microfilaments function. G-actin can be irreversibly aggregated into F-actin which blocks the cell surface pseudopodia formation. TGFB1 up regulated the CFL1, G-actin, and F-actin expressions which resulted in microfilament polymerization. Therefore, it was concluded that CFL1 promotes the EMT process and gastric tumor cell invasion through cytoskeletal rearrangement [63]. Hyaluronic acid (HA) as an ECM component is mainly secreted by fibroblasts in response to humoral signals of tumor cells [166]. HMMR is an intracellular HA binding protein belonging to the microtubule-associated protein (MAP) family which is associated with microfilament formation and cell movement in tumor cells. An expressional study has been performed on the levels of HMMR protein expressions in GC patients and showed positive expression in cellular mucosa and cytoplasm in tumor cells, while it was expressed rarely in normal gastric mucosa. There was also a significant converse correlation between HMMR expression and lymph node involvement [64].

Microfibril associated protein 2 (MFAP2) is one of the main regulators of microfibril function which binds with ECM and TGF-β through specific domains in carboxyl and amino-terminal, respectively [167, 168]. It has been reported that there was MFAP2 up regulation in GC tissues which was associated with depth of invasion, lymph node involvement, tumor stage, and poor survival. Moreover, MFAP2 induces the EMT process and gastric tumor cell proliferation through TGF-β/SMAD2/3 signaling pathway. The MFAP2 knockdown significantly modulated the EMT factors in which Vimentin and Snail expressions were decreased while E-cadherin was up regulated [65]. Spondin 2 (SPON2) is a secreted ECM protein belonged to the F-spondin family which is an integrin ligand [169]. It has been observed that there was SPON2 increased expression in GC tissues in comparison with the normal margins. There were significant correlations between SPON2 up regulation, grade of tumor differentiation, depth of invasion, lymph node involvement, and advanced stages in GC cases. The cases with SPON2 over expression had lower survival compared with under expressed GC patients. The tumors with advanced depth of invasion and metastatic lymph nodes had increased levels of SPON2 expression. Moreover, they showed that there was a direct association between SPON2 and MMP9 expressions among GC patients [66].

### Matricellular proteins and growth factors

In this section, we summarize seven matricellular protein gene products including secreted protein acidic and cysteine rich (SPARC), SPARC like protein 1 (SPARCL1), serpin family E member 1 (SERPINE1), fibroblast growth factor receptor 2 (FGFR2), thrombospondin 1 (THBS1), latent transforming growth factor beta binding protein 2 (LTBP2), and periostin (POSTN).

Matricellular proteins are ECM glycoproteins that includes osteopontin, tenascins, POSTN, SPARC, and thrombospondins that are associated with embryogenesis, stem cell niches, and tissue remodeling [79, 170]. Most of them regulate cell–matrix adhesions via binding with fibronectin or collagen. SPARC is a matricellular protein produced by endothelial cells which regulates cell–matrix interactions during tissue remodeling through binding with ECM components such as collagens and fibronectin [171, 172]. It has been shown that there was increased expression of SPARC in GC tissues in comparison with normal margins. There were correlations between SPARC up regulation, serosal invasion, poor survival [67]. SERPINE1 and SPARC over expressions were also observed in GC tissues which were correlated with poor survival [68]. SPARC like protein 1 (SPARCL1) is a matricellular protein involving in tumor progression and tissue regeneration [173]. It has been reported that there was decreased expression of SPARCL1 in GC tissues compared with normal margins. There were significant correlations between SPARCL1 down regulation, poor prognosis, survival, histological type, and tumor size. There was also an association between SPARCL1 expression and grade of tumor differentiation in which there was a significant declining levels of expression toward the poorly differentiated tumors [69]. The thrombospondin is a calcium binding glycoprotein involving in wound healing and angiogenesis in which it inhibits angiogenesis via interaction with CD36 on endothelial cells and promoting apoptosis [174]. Fibroblast growth factors (FGFs) are heparin-binding growth factors involving in cell proliferation and angiogenesis through FGF receptors (FGFRs). FGF7 as a keratinocyte growth factor promotes FGFR2 dimerization and initiates various signaling pathways such as MAPK and PI3K/Akt [175, 176]. Thrombospondin 1(THBS1) is an endogenous angiogenesis inhibitor [177] which is associated with FGF7/FGFR2 during GC migration and invasion. It has been shown that there were higher levels of FGFR2 and THBS1 expressions in GC tumors compared with normal margins. There was an association between THBS1 expression and well/moderate differentiation which suggests a less aggressive behavior. Since there was a direct correlation between THBS1 and FGFR2 expressions, the FGF7/FGFR2 may induce GC invasion through THBS1. Moreover, it was shown that the FGF7/FGFR2 up regulates THBS1 through PI3K/Akt/mTOR pathway [70].

LTBP2 is belonged to the ECM proteins which has calcium-binding epidermal growth factor like and TGF-β binding domains [178]. LTBPs have an important role in ECM regulation and TGF-β activation [179]. It has been reported that there were increased expression of LTBP2 in GC tissues and cell lines which was correlated with depth of invasion, lymph node involvement, poor survival, and tumor stage [71]. POSTN is one of the ECM components that has oncogenic role through integrins and EGFR to activate the AKT/PKB and FAK signaling pathways in different tumors [180, 181]. It has been shown that there were lower levels of periglandular POSTN expression in GC tissues compared with normal margins. The metastatic lymph nodes had significantly lower levels of POSTN expression. Moreover, there was an inverse correlation between POSTN expression and tumor stage. It seems that this tumor suppressor role of POSTN can be related to the probable correlation between POSTN, P53, and E-cadherin through Rb/E2F1/p14ARF/MDM2 signaling pathway [72].

### Proteoglycans and extra cellular glycoproteins

In this section, we summarize four matricellular protein gene products including versican (VCAN), asporin (ASPN), lumican (LUM), and mucin 2 (MUC2).

The ECM is composed of various proteins such as proteoglycans (PGs) and collagens that organize physiological and pathological functions [182]. Proteoglycans are glycosylated proteins spreading throughout the mammalian tissues which are associated with tissue remodeling, cellular adhesion, and signaling pathways. VCAN as a chondroitin sulfate PG is mainly accumulated in tumor stroma and has key functions in regulation of cell proliferation, self-renewal, and migration [183, 184]. It has been shown that the GC tissues had significantly higher levels of VCAN expression. Moreover, there was a correlation between VCAN over expression and GC poor prognosis [73].

Asporin (ASPN) is one of the members of small Leucine-rich proteoglycan (SLRP) family that is associated with ECM modification [185]. It has been reported that there was significantly increased levels of ASPN expression in GC tissues compared with normal margins. The migration of gastric tumor cells also significantly decreased following the ASPN knockdown. Moreover, it seems that there were some correlations between CD44, MMP2, and ASPN expression in which ASPN silencing significantly decreased the levels of CD44 and MMP2 expressions in GC cells. Therefore in can be concluded that the ASPN is involved in regulation of GC migration and metastasis via EGFR-CD44/MMP2 signaling pathway [74]. Cancer-associated fibroblasts (CAFs) are stromal cells characterized by their spindle-shape morphology, fibroblast activation protein alpha, and α-smooth muscle actin expressions [186]. These cells are associated with tumor progression via secretion of several factors such as cytokines and growth factors [187]. LUM is an external proteoglycan belonged to the SLRPs family. It seems that the LUM exerts its role in gastric tumor progression through integrin β1-FAK signaling pathway. FAK as a tyrosine kinase is activated by integrin or growth factor receptors and has key role in regulation of cyclins and signaling pathways such as PI3K/AKT and MAPK [188, 189]. It has been observed that there was LUM up regulation in gastric CAFs which was directly correlated with advanced stage, poor survival, depth of invasion, and lymph node involvement. LUM knockdown in CAFs also suppresses the GC cell proliferation, migration, and in vivo tumorigenesis [75].

Mucins are glycoproteins comprising of a mucin core and O-linked oligosaccharides produced by epithelial tissues which are involved in epithelial surfaces protection, inflammation, renewal, and tumorigenesis [190]. Although, expressions of MUC1, MUC5AC, and MUC6 are observed in normal gastric mucosa [191], there is not any MUC2 expression in normal gastric mucosa [192]. It has been observed that majority of GC tissues had MUC2 expression. Moreover, MUC2 reactivity was directly correlated with grade of differentiation [76]. Sialic acids are monosaccharides that are attached to the membranous glycoproteins and glycolipids. It has been shown that there was a correlation between tumor invasion and deregulation of terminal 2, 3-linked sialic acids [193, 194]. The up regulation of 2, 3-linked sialic acid residues were associated with lymph node involvement and depth of tumor invasion. MAL as a specific sialic acid-binding lectin was observed in gastric cancerous regions, whereas it was not detected in normal margins [195].

## Conclusion

Regarding the importance of extra cellular matrices and components in GC metastasis and invasion, in present review we summarized 48 of significant ECM related factors which have been reported among GC patients. It has been shown that the intra cellular signaling pathways associated with tumor invasion also exert their role through regulation of local ECM. Therefore, according to the lower complexity of ECM compared with intracellular signaling pathways, the ECM can be a more reliable tumor therapeutic target with lower side effects. Indeed this review paves the way of introducing an extra cellular based prognostic and diagnostic panel marker for the GC patients.

## Data Availability

The datasets used and/or analyzed during the current study are available from the corresponding author on reasonable request.

## Abbreviations

ANOS1: Anosmin 1
ASPN: Asporin
CAFs: Cancer-associated fibroblasts
CFL1: Cofilin 1
COX2: Cyclooxygenase 2
CTHRC1: Collagen triple helix repeat containing 1
ECM: Extracellular matrix
ECM1: Extracellular matrix protein 1
EMMPRIN: Extracellular MMP inducer
EMT: Epithelial-mesenchymal transition
FGFs: Fibroblast growth factors
FGFRs: FGF receptors
FN: Fibronectin
FNDC1: Fibronectin type III domain containing 1
GC: Gastric cancer
GLI1: GLI family zinc finger 1
HA: Hyaluronic acid
HMMR: Hyaluronan mediated motility receptor HPSE Heparanase
LAMC2: Laminin subunit gamma 2
LGALS1: Galectin 1
LUM: Lumican
MAP: Microtubule associated protein
MFAP2: Microfibril associated protein 2
MMPs: Matrix metalloproteinases
MMRN2: Multimerin 2
PCDH: Protocadherins
PGs: Proteoglycans
POSTN: Periostin
SLRP: Small Leucine-rich proteoglycan
SPON2: Spondin 2
SPARCL1: SPARC like protein 1
TFPI2: Tissue factor pathway inhibitor 2
THBS1: Thrombospondin 1
TIMPs: Tissue inhibitors of metalloproteinases
VCAN: Versican
VEGF: Vascular endothelial growth factor
